# Melatonin, but not melatonin receptor agonists Neu-P11 and Neu-P67, attenuates TNBS-induced colitis in mice

**DOI:** 10.1007/s00210-016-1214-x

**Published:** 2016-02-22

**Authors:** Marta Zielińska, Agata Jarmuż, Maciej Sałaga, Radzisław Kordek, Moshe Laudon, Martin Storr, Jakub Fichna

**Affiliations:** Department of Biochemistry, Medical University of Lodz, Lodz, Poland; Department of Pathology, Faculty of Medicine, Medical University of Lodz, Lodz, Poland; Neurim Pharmaceuticals LTD, Tel-Aviv, Israel; Walter Brendel Center of Experimental Medicine, University of Munich, Munich, Germany; Department of Medicine, Division of Gastroenterology, Ludwig Maximilians University of Munich, Munich, Germany

**Keywords:** Colitis, Inflammatory bowel diseases, Melatonin, Melatonin receptor agonists

## Abstract

Melatonin is known as a strong antioxidant and possesses anti-inflammatory properties. Recently, melatonin was shown to improve colitis in animal models of inflammatory bowel diseases. The aim of the present study was to characterize the role of melatonin receptors (MT) in the anti-inflammatory effect of melatonin and to assess the anti-inflammatory potential of two novel MT receptor agonists, Neu-P11 and Neu-P67, in the mouse model of trinitrobenzenesulfonic acid (TNBS)-induced colitis. Colitis was induced on day 1 by intracolonic (i.c.) administration of TNBS in 30 % ethanol in saline. Melatonin (4 mg/kg, per os (p.o.)), Neu-P11 (20 mg/kg, p.o.; 50 mg/kg, intraperitoneally (i.p.), 50 mg/kg, i.c.), and Neu-P67 (20 mg/kg, p.o.) were given twice daily for 3 days. Luzindole (5 mg/kg, i.p.) was injected 15 min prior to melatonin administration. On day 4, macroscopic and microscopic damage scores were assessed and myeloperoxidase (MPO) activity quantified using O-dianisidine-based assay. Melatonin significantly attenuated colitis in mice, as indicated by the macroscopic score (1.90 ± 0.34 vs. 3.82 ± 0.62 for melatonin- and TNBS-treated mice, respectively), ulcer score (0.87 ± 0.18 vs. 1.31 ± 0.19, respectively), and MPO activity (4.68 ± 0.70 vs.6.26 ± 0.94, respectively). Luzindole, a MT receptor antagonist, did not inhibit the anti-inflammatory effect of melatonin (macroscopic score 1.12 ± 0.22, ulcer score 0.50 ± 0.16); however, luzindole increased MPO activity (7.57 ± 1.05). MT receptor agonists Neu-P11 and Neu-P67 did not improve inflammation induced by TNBS. Melatonin, but not MT receptor agonists, exerts potent anti-inflammatory action in acute TNBS-induced colitis. Our data suggests that melatonin attenuates colitis by additional, MT receptor-independent pathways.

## Introduction

Melatonin is a hormone produced from tryptophan mainly by the pineal gland, but also in peripheral organs, including immune cells and the gastrointestinal (GI) tract (Chen et al., 2011), where it is secreted by enterochromaffin cells of the GI mucosa. The concentration of melatonin in the GI tract is approximately 400 higher than in the pineal gland. Melatonin exerts multiple activities, including antioxidative, immunomodulatory, and anti-inflammatory (Bubenik, 2008). Consequently, melatonin has been regarded as a strong antioxidant and scavenger of peroxynitrite, peroxyl, oxygen, and superoxide anion radicals.

The primary mechanism of melatonin action is stimulation of melatonin receptors (MT), which belong to the G protein-coupled receptor family (Pandi-Perumal et al., 2008). In mammals, two types of MT receptors have been identified: Mel_1a_ and Mel_1b_, later renamed MT1 and MT2. MT receptor activation leads to inhibition of adenylyl cyclase activity and causes decrease in cAMP concentration. In addition, MT2 receptor-dependent signaling also involves cGMP-related pathways.

In the GI tract, MT receptors were found in the mucosa, submucosa, muscle cells, and myenteric plexus. The highest expression of MT receptors was detected in the small intestine, duodenum, and colon (Poirel et al., 2003). In humans, MT2 receptors are widely distributed in epithelial and endocrine cells as well as submucosal and myenteric plexuses of the large intestine. In the small intestine, the MT2 expression in the submucosal and myenteric plexuses and in the epithelial cells is strong in comparison with MT1 receptors. Moreover, MT1 receptors are not located on the vascular and endocrine cells of the small intestine (Soderquist et al., 2015).

MT receptors have also been located on the membrane of immune cells (e.g., CD4 T, CD8 T, and B lymphocytes) (Garcia-Maurino et al., 2000a; Lardone et al., 2010), and melatonin was found to regulate both cellular and humoral response via MT receptors (Drazen and Nelson, 2001). Through MT receptors, melatonin regulates the activation of transcriptional factors involved in immunological responses as well as immune cell differentiation and proliferation (Morgan et al., 1995). It has also been reported that the proliferation of T cells increases in mice treated with melatonin (Lin et al., 2013).

Lately, it was proposed that the impairment in the release and distribution of endogenous melatonin may be involved in the pathogenesis of inflammatory bowel diseases (IBD): Crohn’s disease and ulcerative colitis (Cuzzocrea et al., 2001b; Mauriz et al., 2013). In line, it was evidenced that melatonin improves colitis in numerous animal models of IBD (Chung et al., 2014; Jena and Trivedi, 2014; Tasdemir et al., 2013; Trivedi and Jena, 2013). For example, melatonin attenuated colitis induced by intracolonic instillation of dinitrobenzenesulfonic acid (DNBS) in rats (Cuzzocrea et al., 2001a). The anti-inflammatory effect was observed in an improved colonic architecture and decreased infiltration of immune cells; the myeloperoxidase (MPO) level was also significantly decreased. Moreover, melatonin inhibited oxidative stress, as indicated by a reduced level of malonyl dialdehyde (MDA) and prevented the upregulation of adhesion molecules, ICAM-1 and P-selectin, in the colon. Finally, alleviation of COX-2 and iNOS expression was observed. In another study, melatonin prevented lipid peroxidation in DNBS-induced colitis, assessed by thiobarbituric acid reactive substances (TBARS) levels, confirming its antioxidative activity (Esposito et al., 2008). Melatonin also reduced colonic inflammatory injury through downregulation of pro-inflammatory molecules, e.g., TNF-α, in a NF-κB-dependent manner (Li et al., 2005), inhibition of c-Jun, phosphorylation, and MMP-2 and MMP-9 activity and expression (Esposito et al., 2008).

Noteworthy, in most of the published studies, melatonin exerted an antioxidative effect and improved microcirculation in the intestinal epithelium (Reiter et al., 2003).

Recently, two novel MT receptor agonists, Neu-P11 and Neu-P67, were developed (She et al., 2009). Both compounds display high affinity at MT receptors and prolonged duration of action. In addition, Neu-P11 has potent antinociceptive effect after per os (p.o.) administration (Chen et al., 2011) and significantly inhibits GI motility (unpublished results). The aim of this study was to compare the possible anti-inflammatory effect of Neu-P11 and Neu-P67 with melatonin and to characterize the involvement of MT receptors in the course of the intestinal inflammation in the mouse model of trinitrobenzenesulfonic acid (TNBS)-induced colitis.

## Materials and methods

### Animals

Male balb/c mice (Animal Facility of Nofer Institute of Occupational Medicine, Lodz, Poland), weighing 24–26 g, were used in the experiments. Mice were housed at a constant temperature (22–23 °C) and maintained under a 12-h light/dark cycle with the access to laboratory chow and tap water.

The study was carried out in strict accordance with the institutional recommendations. The protocol was approved by the Local Ethical Committee for Animal Experiments (# 589/2011).

### Drugs

Melatonin and luzindole were purchased from Tocris Bioscience (Ellisville, MO, USA). Neu-P11 and Neu-P67 were obtained from Neurim Pharmaceuticals Ltd., Israel. All other reagents, unless otherwise stated, were purchased from Sigma-Aldrich (Poznan, Poland).

### Pharmacological treatments

Melatonin, Neu-P11, Neu-P67, and luzindole were dissolved in 5 % dimethyl sulfoxide (DMSO) in saline. Animals without treatment received vehicle alone (5 % DMSO in saline). Melatonin was administered p.o. at the dose of 4 mg/kg; Neu-P11 was given p.o. (20 mg/kg), i.p. (50 mg/kg), or i.c., 50 mg/kg; and Neu-P67 was administered p.o. (20 mg/kg). Luzindole was administered i.p. at the dose of 5 mg/kg 15 min prior to melatonin injection. The first treatment with the drug (melatonin, Neu-P11, NeuP-67, luzindole) was 15 min before the induction of colitis with TNBS.

All drugs were given twice daily from day 1–day 3 and prepared before injections. The doses of all drugs used in our experiments were selected based on our preliminary studies and available literature (Chen et al., 2011; Trivedi and Jena, 2013).

### Induction of colitis and assessment of colonic damage

Colitis was induced by i.c. injection of trinitrobenzenesulfonic acid (TNBS) on day 1, as described previously (Sobczak et al., 2014b). Briefly, mice (*n* = 6–8 per group) were anesthetized with isoflurane (Aerrane, Baxter, Deerfield, USA) and TNBS (4 mg in 0.1 ml of 30 % ethanol in saline) was injected into the distal colon (2.5 cm proximally to the anus) using a catheter. Mice were weighted daily and monitored for clinical symptoms of colitis, including diarrhea and bloody stool. On day 4, animals were sacrificed and the total macroscopic score was assessed. The total macroscopic score is a sum of the following parameters: for ulcer score 0.5 points for each 0.5 cm, adhesion (0–2), wall thickness measured in millimeters, presence of hemorrhage; fecal blood and diarrhea increased the score by 1 point for each additional feature.

Moreover, samples for assessment of MPO activity and for histology were collected and kept in −80 °C and 4 °C, respectively.

### Determination of tissue myeloperoxidase activity

To monitor the degree of inflammation, isolated colon sections (15–30 mg) were washed and homogenized in hexadecyltrimethylammonium bromide (HTAB) buffer (0.5 % HTAB in 50 mM potassium phosphate buffer, pH 6.0; 50 mg of tissue/ml) using Ika Ultra Turrax Disperser T25 Digital 2 (Sigma-Aldrich, Poznan, Poland). Homogenates were centrifuged (15 min, 13.200×*g*, 4 °C), and the supernatants were collected to new tubes. Next, 7 μl of supernatant was added on a 96-well plate, followed by 200 μl of 50 mM potassium phosphate buffer (pH 6.0), containing 0.167 mg/ml of O-dianisidine hydrochloride and 0.05 μl of 1 % hydrogen peroxide. Absorbance was measured at 450 nm after 30 and 60 s (iMARK Microplate Reader, Biorad, UK). MPO activity was expressed in milliunits per gram of wet tissue, as described earlier (Sobczak et al., 2014a). MPO assay is a sensitive test and therefore to obtain representative results, all measurements were performed in triplicate for 6–8 colon sections collected from all animals in the group.

### Histology

Distal colon sections were stapled flat, mucosal side-up, onto cardboard strips and fixed in 4 % formalin for >24 h at 4 °C. Samples were dehydrated, embedded in paraffin, sectioned at 5 μm, and mounted onto slides. Sections were stained with hematoxylin and eosin and examined using a microscope (Motic AE31, Ted Pella, Sweden). Photographs were taken using a digital imaging system consisting of a digital camera (Moticam 2300, Ted Pella, Sweden) and image analysis software (Motic Images Plus 2.0, Germany).

The microscopic total damage score was assessed as described previously (Galeazzi et al., 1999, with minor modifications), using the following parameters: the extent of muscle thickening, and the presence and degree of immune cell infiltration (normal = 1, moderate = 2, transmural = 3), the destruction of mucosal architecture (normal = 1, moderate = 2, extensive = 3), and the goblet cell depletion and crypt abscesses (presence = 1, absence = 0).

### Statistical analysis

The results are expressed as mean ± standard error of the mean (SEM). Values given with “n” refer to the number of experiments performed in tissues from different animals. Statistical analysis was performed using Prism 5.0 (GraphPad Software Inc., La Jolla, CA, USA). ANOVA followed by Newman-Keuls post hoc testing was used for multiple comparisons. *p* values <0.05 were considered statistically significant.

## Results and discussion

In the present study, we confirmed that melatonin exerts a potent anti-inflammatory action in the mouse model of TNBS-induced colitis. In contrast, Neu-P11, a multi-targeted drug candidate with combined functionality at MT1\MT2 receptors (sleep promoting, neuroprotection) and 5HT1A\D receptors (antidepressant and anxiolytic effects), did not exert anti-inflammatory action in the GI tract. Similarly, Neu-P67, a potent agonist of MT1/MT2 receptors and an inhibitor of fatty acid amide hydrolase (FAAH), an enzyme involved in degradation of endocannabinoids, also failed to attenuate colonic inflammation.

**Fig. 1:**
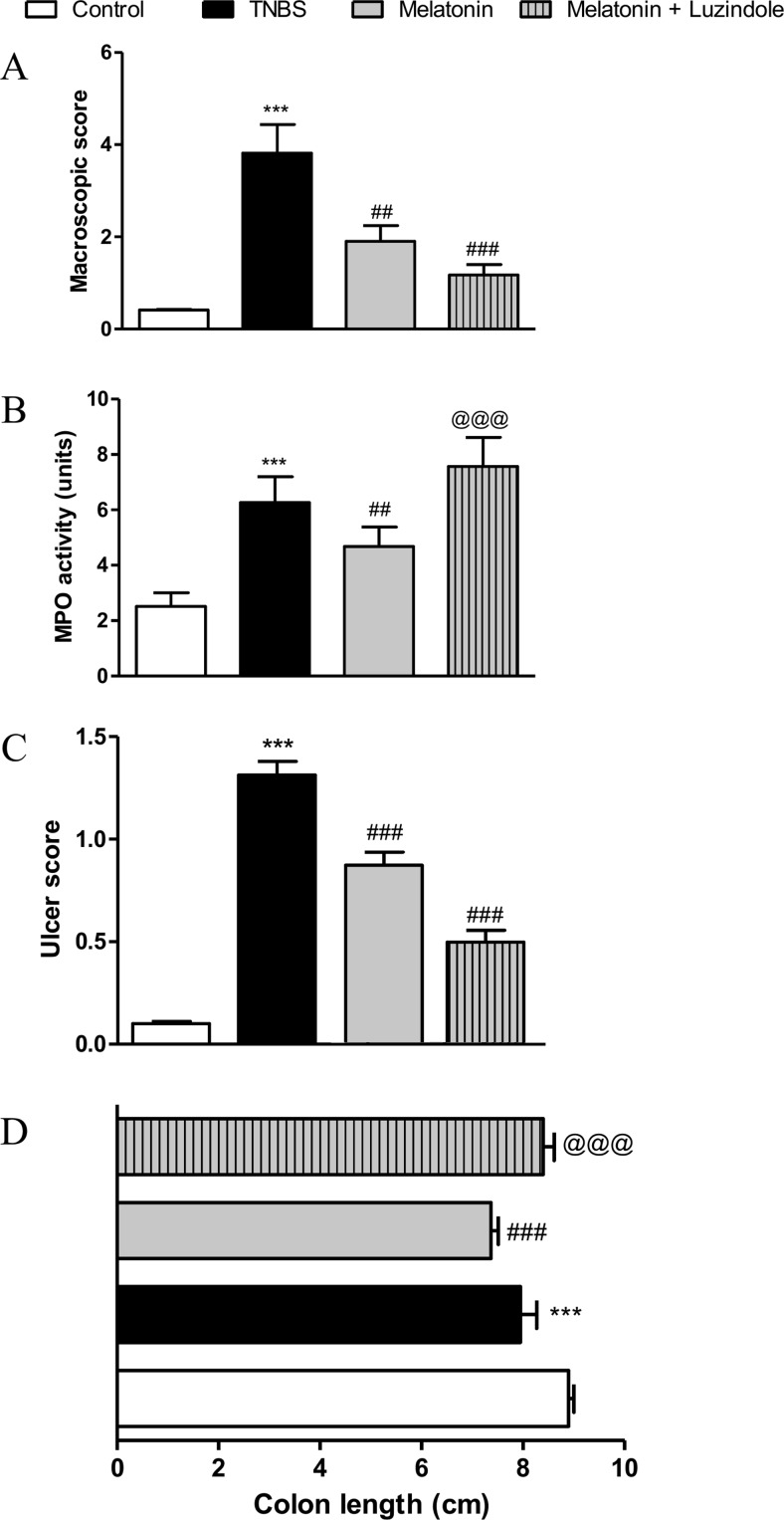
Oral administration of melatonin at the dose of 4 mg/kg twice daily significantly attenuated colitis in mice but blockage of MT receptors did not cause worsening of the intestinal inflammation. Figure shows data for macroscopic score (**a**), MPO activity (**b**), ulcer score (**c**), and colon length (**d**). ***p < 0.001 as compared with control, ##*p* < 0.01, ###*p* < 0.001 as compared with TNBS-treated animals, @@@ as compared with melatonin-treated mice. Data represent mean ± SEM, *n* = 6–8 mice per group

As shown in Fig. 1, melatonin given p.o. at the dose of 4 mg/kg twice daily significantly improved colitis in mice, as indicated by the macroscopic score (1.90 ± 0.34 vs. 3.82 ± 0.62 for TNBS-treated mice) and ulcer score (0.87 ± 0.18 vs. 1.31 ± 0.19). Noteworthy, administration of melatonin decreased MPO activity (4.67 ± 0.70 vs. 6.26 ± 0.94). In the colon, tissue injury progression occurs because of leukocyte infiltration and release of free oxygen radicals from activated immune cells, which further induce pro-inflammatory cytokine production. MPO expressed in the immune cells, in the presence of Cl^−^ converts hydrogen peroxide to hypochloric acid (HOCl), which is a potent oxidant and antimicrobial agent, and plays important role in immune response. Since MPO activity in the colon is linearly related to the infiltration of immune cells, it is well established for quantification of colitis.

Body weight is another important parameter in the development of colitis. In this study, loss of weight was observed in animals after TNBS instillation, but not in the control group. Furthermore, a less significant loss of body weight and a quicker recovery was noted in mice treated with melatonin (data not shown). Our results are consistent with data obtained by Cuzzocrea et al. (Cuzzocrea et al., 2001a), who evidenced that the anti-inflammatory effect of melatonin was observed through an improved colonic architecture, decreased infiltration of immune cells, and significantly lower MPO level.

**Fig. 2:**
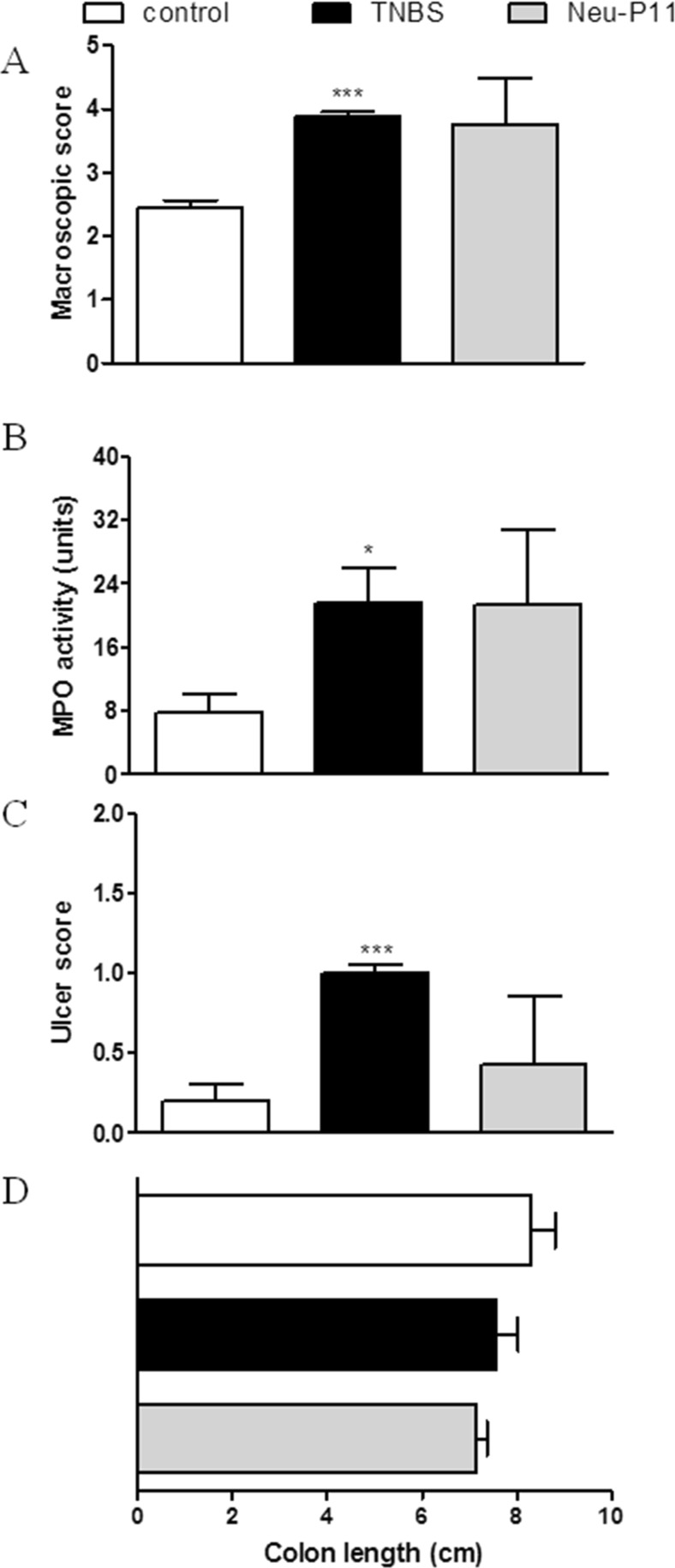
Oral administration of Neu-P11 at the dose of 20 mg/kg twice daily did not improve colitis in mice. Figure shows data for macroscopic score (**a**), MPO activity (**b**), ulcer score (**c**), and colon length (**d**). *p < 0.05, ***p < 0.001 as compared with control. Data represent mean ± SEM, *n* = 6–8 mice per group

**Fig. 3:**
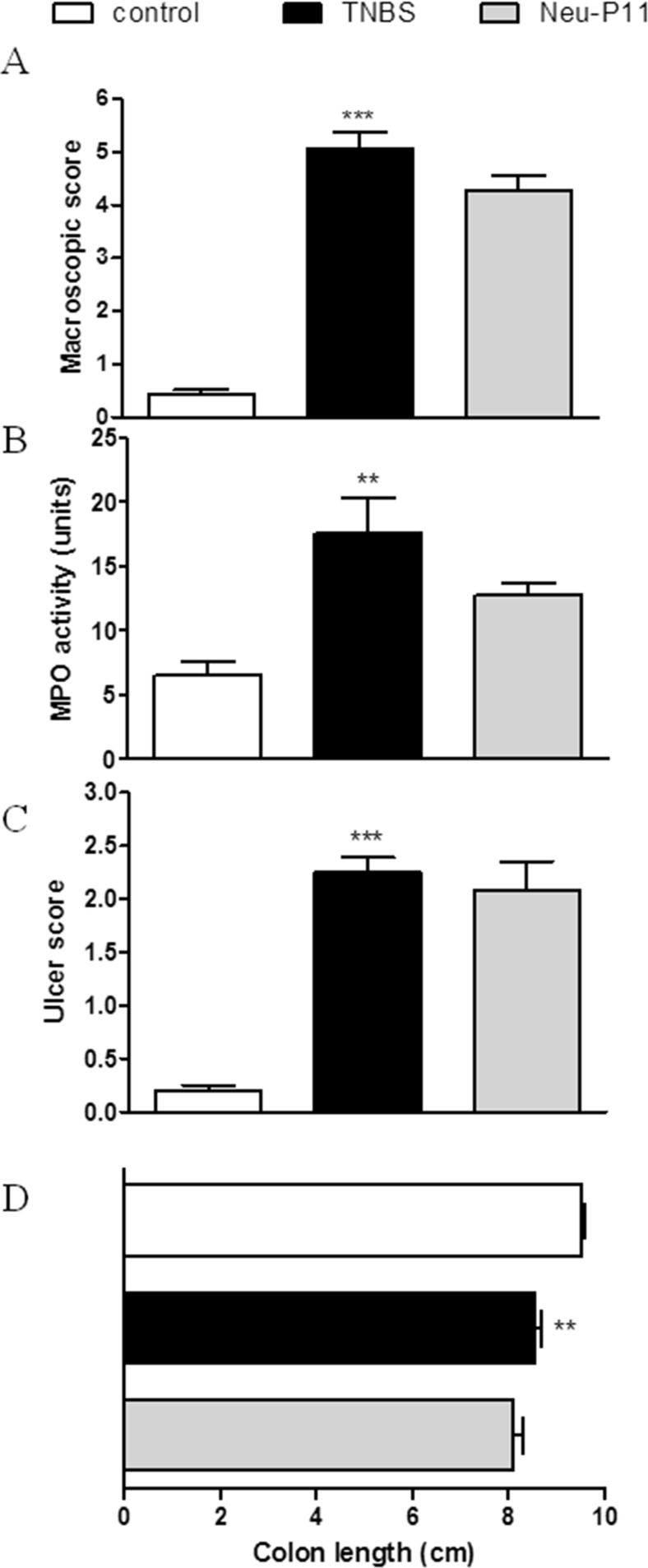
Intraperitoneal injection of Neu-P11 at the dose of 50 mg/kg twice daily did not improve colitis in mice. Figure shows data for macroscopic score (**a**), MPO activity (**b**), ulcer score (**c**), and colon length (**d**). **p < 0.01, ***p < 0.001 as compared with control. Data represent mean ± SEM, *n* = 6–8 mice per group

**Fig. 4:**
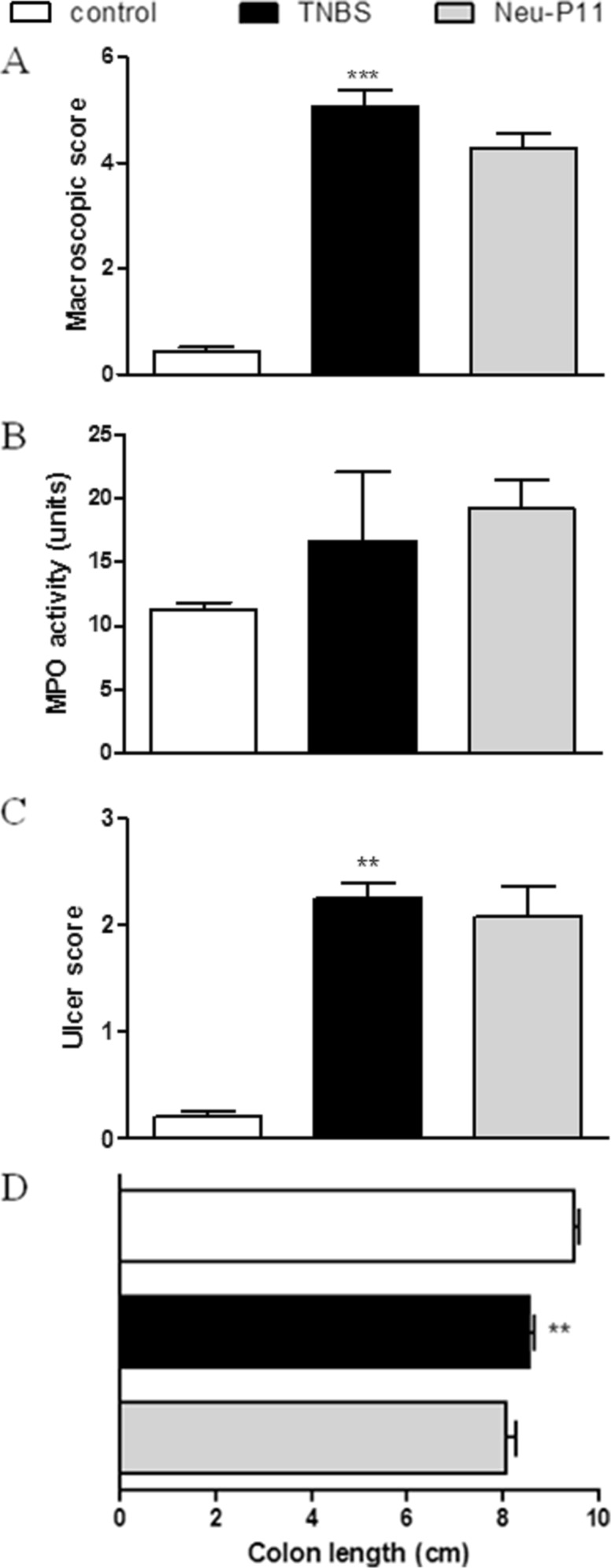
Intracolonic injection of Neu-P11 at the dose of 50 mg/kg twice daily did not improve colitis in mice. Figure shows data for macroscopic score (**a**), MPO activity (**b**), ulcer score (c), and colon length (**d**). **p < 0.01, ***p < 0.001 as compared with control. Data represent mean ± SEM, *n* = 6–8 mice per group

Consequently, we hypothesized that Neu-P11, which displays high binding affinity at MT receptors and has good oral bioavailability, should also improve colitis. However, we observed that Neu-P11 did not attenuate colitis in mice after p.o. administration at the dose of 20 mg/kg (twice daily), as shown by the macroscopic score (3.76 ± 0.72 vs. 3.88 ± 0.08 for TNBS-treated mice), MPO activity (21.35 ± 9.54 vs. 21.62 ± 4.26), and the ulcer score (0.43 ± 0.43 vs. 1.00 ± 0.01, *p* = 0.24; Fig. 2). To further investigate its effect in the inflamed colon, Neu-P11 was injected i.p. and i.c. (both 50 mg/kg, twice daily). We observed no anti-inflammatory action of Neu-P11 administered i.p. (macroscopic score 4.28 ± 0.27 vs. 5.07 ± 0.29, MPO activity 12.72 ± 0.96 vs. 17.57 ± 2.75, ulcer score 2.08 ± 0.27 vs. 2.25 ± 0.14 for Neu-P11 vs. TNBS-treated mice, respectively; Fig. 3) or i.c. (macroscopic score 4.28 ± 0.27 vs. 5.07 ± 0.29, *p* = 0.08, MPO activity 19.19 ± 2.27 vs. 16.69 ± 5.42, ulcer score 2.08 ± 0.27 vs. 2.25 ± 0.14 for Neu-P11 vs. TNBS-treated mice, *p* = 0.65, respectively; Fig. 4).

**Fig. 5:**
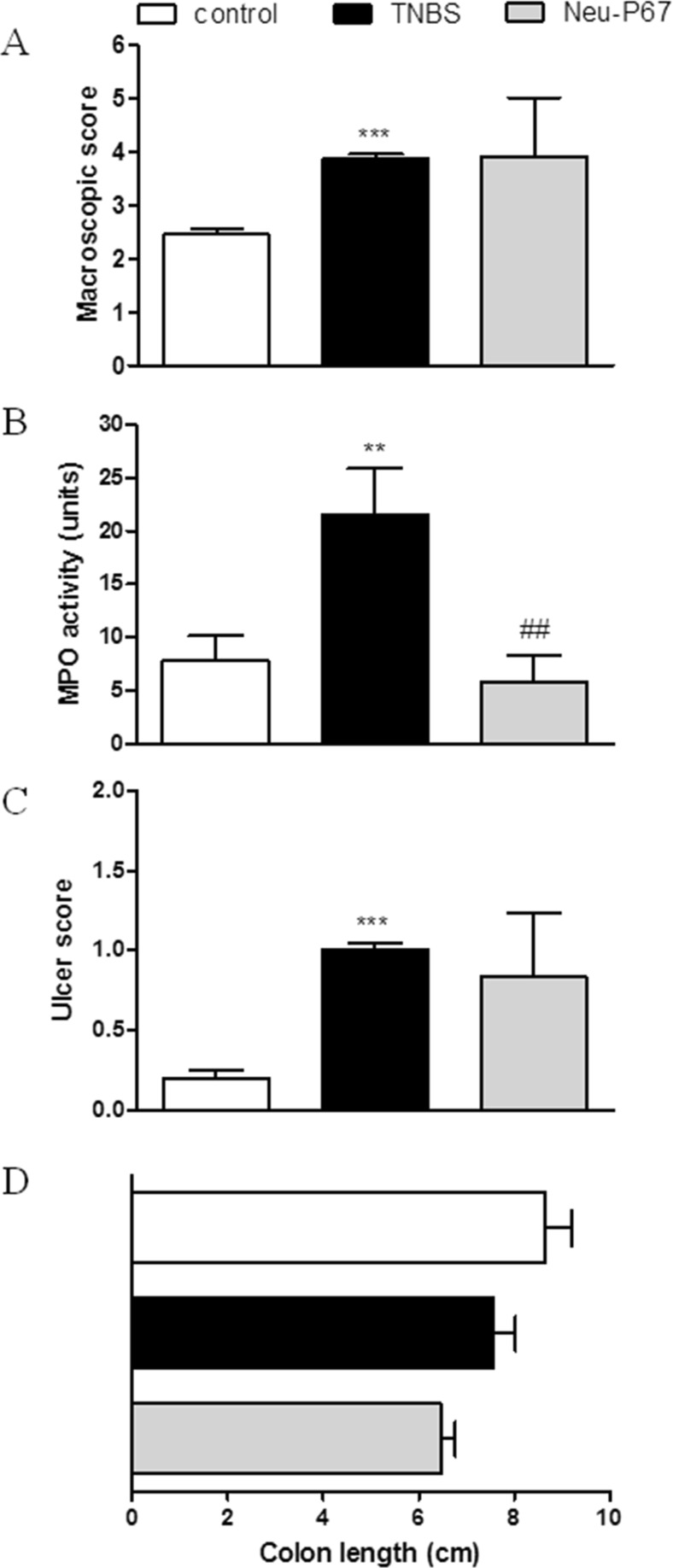
Oral administration of Neu-P67 at the dose of 20 mg/kg twice daily did not improve macroscopic score, but decreased MPO activity in TNBS-treated mice. Figure shows data for macroscopic score (**a**), MPO activity (**b**), ulcer score (**c**), and colon length (**d**). **p < 0.01, ***p < 0.001 as compared with control, ##*p* < 0.01, as compared with TNBS-treated animals. Data represent mean ± SEM, *n* = 6–8 mice per group

Earlier reports showed that the inhibition of FAAH significantly attenuated inflammation in several animal models of colitis (Salaga et al., 2014), what encouraged us to use Neu-P67, a novel mixed MT receptor agonist—FAAH blocker in this study. However, Neu-P67 did not alleviate colitis in mice after p.o. administration (macroscopic score 3.92 ± 1.10 vs. 3.88 ± 0.08, ulcer score 0.83 ± 0.40 vs. 1.00 ± 0.00 for Neu-P67 and TNBS-treated mice, respectively; Fig. 5a–b). Surprisingly, Neu-P67 significantly decreased MPO activity (5.85 ± 2.40 vs. 21.62 ± 4.26 for TNBS and Neu-P67 vs. TNBS alone-treated mice, respectively, *p* = 0.008; Fig. 5C). This observation was not in line with macro- and microscopic analysis, but remained consistent throughout replicates. It suggests that Neu-P67 affects the immune cell infiltration; moreover, possibly non-MT receptor-dependent mechanisms, e.g., involving inhibition of FAAH, are very likely to play a role in the decrease of MPO activity observed in this study. However, further examination is required to understand this process.

**Fig. 6:**
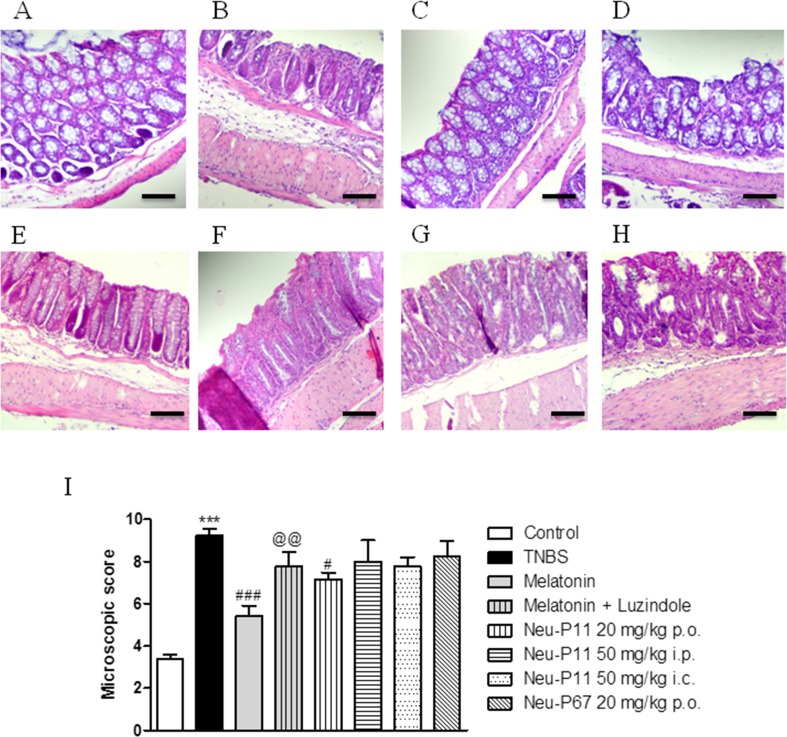
Melatonin attenuated microscopic damage score. Microscopic total damage score and representative micrographs of hematoxylin and eosin-stained sections of distal colon from **a** control, **b** TNBS, **c** TNBS and melatonin (4 mg/kg, p.o., twice daily), **d** TNBS and melatonin (4 mg/kg, p.o., twice daily) co-administered with luzindole (5 mg/kg, i.p.), **e** TNBS and Neu-P11 (20 mg/kg, p.o., twice daily), **f** TNBS and Neu-P11 (50 mg/kg, i.p., twice daily), **g** TNBS and Neu-P11 (50 mg/kg, i.c., twice daily) treated mice, (H) TNBS and Neu-P67 (20 mg/kg, p.o., twice daily), and **i** total microscopic damage score. *Scale bar* = 100 μm. ***p < 0.001, as compared with control. #*p* < 0.05, ###*p* < 0.001, as compared with TNBS-treated mice. @@*p* < 0.01, as compared with melatonin-treated mice. Data represent mean ± SEM, *n* = 6–8 mice per group

The macroscopic scoring was consistent with microscopic imaging. As shown in Fig. 6, the architecture of the colon in TNBS-treated mice was disrupted; the tissue was infiltrated with immune cells, the muscle layer was thicker, and the mucosal layer severely damaged compared with control. The microscopic parameters were improved after melatonin (4 mg/kg, p.o., twice daily; Fig. 6C), but not Neu-P11 (20 mg/kg, p.o.; Fig. 6D; 50 mg/kg i.p.; Fig. 6E; 50 mg/kg i.c.; Fig. 6F) or Neu-P67 (20 mg/kg, p.o., twice daily; Fig. 6G) treatment.

Because we did not observe any anti-inflammatory activity of novel MT receptor agonists Neu-P11 and Neu-P67, we decided to verify the role of MT receptors in the anti-inflammatory effect of melatonin. We thus used luzindole, a competitive MT receptor antagonist (Dubocovich et al., 1998). Surprisingly, luzindole did not block the action of melatonin, as indicated by macroscopic and ulcer score (1.90 ± 0.34 and 0.87 ± 0.18 for melatonin-treated group vs. 1.17 ± 0.22 and 0.50 ± 0.16 for melatonin + luzindole-treated group), Fig 1. Luzindole did not improve MPO activity (4.68 ± 0.70 for melatonin-treated group vs. 7.57 ± 1.05 for melatonin + luzindole-treated group) and microscopic damage score (5.40 ± 0.51 for melatonin-treated group vs. 7.75 ± 0.70 for melatonin + luzindole-treated group). Of note, luzindole slightly improved macroscopic scoring in mice, what suggests that melatonin has a great anti-inflammatory potential even when MT receptors are blocked. Interestingly, luzindole increased MPO level, suggesting that modulation of leukocyte recruitment could be partially mediated by MT2 receptors (luzindole exerts 15–25-fold higher selectivity for MT2 than MT1 receptors). However, development of highly selective MT1 and MT2 antagonists would help to better understand the role of MT receptors in physiological and pathophysiological processes.

Collectively, our data suggests that the activation of MT receptors, which is sufficient to produce antinociception and potent inhibition of GI motility, does not produce anti-inflammatory effect in the colon. Consequently, pathways other than MT receptor-dependent may be involved in the anti-inflammatory action of melatonin. This is in line with a study by Lahiri et al. (Lahiri et al., 2009), in which melatonin at the doses ranging from 10 to 40 mg/kg injected i.p. exerted protective role in reflux esophagitis. Melatonin-induced effect on antioxidant parameters (MDA, SOD, and GSH) and expression of pro-inflammatory molecules, including TNF-α, IL-1β, and IL-6 levels in the colon, was not reversed by luzindole. These results suggest a MT receptor-independent action, what is consistent with our observations (Lahiri et al., 2009).

Despite a number of additional experiments, we did not manage to identify the non-MT receptor-related mechanism of action of melatonin. Earlier studies suggest that melatonin can bind with nuclear binding receptors, namely retinoid orphan receptors/retinoid Z receptors (ROR/RZR), located on lymphocytes and monocytes (Garcia-Maurino et al., 2000b). Through this pathway, melatonin is involved in the regulation of immunological processes and influences lymphocyte T maturation (Smirnov, 2001). Moreover, melatonin was a potent inhibitor of the expression of 5-lipoxygenase in human B cells, an important enzyme involved in allergy and inflammation also through ROR/RZR (Missbach et al., 1996).

Furthermore, melatonin binds to calmodulin and calreticulin, involved in the cytoskeleton regulation and control of nuclear receptors (Pedrosa et al., 2010). Calmodulin and calreticulin inhibit protein activation, followed by protein distribution and cell cycle disruption (Blask et al., 2002). Another possible binding site for melatonin is quinone reductase 2, which participates in the protection against oxidative stress by preventing electron transfer reactions of quinones (Nosjean et al., 2000).

Kang et al. showed that melatonin attenuated the increased expression of TLR at protein level after ischemia and reperfusion injury (Kang et al., 2011). Moreover, melatonin decreased TNF-α, IL-6, and iNOS levels, but also triggered MAPK and NF-κB. The inhibitory action of melatonin may be thus combined with MyD88 signaling of the TLR system (Kang et al., 2011).

The antioxidative properties of melatonin could also play a role in its anti-inflammatory effect (Zhang and Zhang, 2014). Namely, melatonin activates antioxidative enzymes, e.g., superoxide dismutase, catalase and glutathione peroxidase, glutathione reductase (GSH-Rd), and glucose-6-phosphate dehydrogenase (G6PD). Moreover, melatonin inhibits the expression of inducible nitric oxide syntase (iNOS). Eventually, melatonin directly influences hydroxyl radical, hydrogen peroxide, nitric oxide, and singlet oxygen secretion (Dziegiel et al., 2003).

Finally, the anti-inflammatory potential of melatonin may be associated with the action of its metabolites (Galano et al., 2013). MT agonists, Neu-P11, and Neu-P67 are not metabolized to the same products as melatonin. Melatonin can be processed in the liver to 6-hydroxymelatonin and further to 6-hydroxymelatonin glucuronide or 6-sulphomelatonin; an alternate metabolic pathway includes formation of N^1^-acetyl-N^2^-formyl-5-metoxy-kynuramine (AMFK) or more stable N^1^-acetyl-5-metoxy-kynuramine (AMK), which share the antioxidative and anti-inflammatory properties with the substrate (Konturek et al., 2007). Interestingly, AMFK and AMK are more potent antioxidants than melatonin against nitric oxide (Galano et al., 2013), and AMK possesses the highest antioxidative activity of all three (Ressmeyer et al., 2003).

## Conclusions

Taken together, our data indicate that the anti-inflammatory effect of melatonin in the colon is independent of MT receptors. Based on earlier reports, we suggest that the most likely mechanism of action may be associated with antioxidant properties of melatonin. In addition, since we observed no aggravation of TNBS-induced colitis upon treatment with novel MT receptor agonists Neu-P11 and Neu-P67, they may be safely used in other (e.g., functional) GI diseases as well as to promote sleep and as antidepressant and anxiolytic agents.
